# Editorial: Mindfulness-based interventions for substance use disorders among minoritized populations

**DOI:** 10.3389/fpsyg.2025.1625381

**Published:** 2025-07-17

**Authors:** Vanessa Caridad Somohano, Alicia E. Vasquez, Josh B. Kaplan, Charles Samuel Henry Robinson

**Affiliations:** ^1^Center to Improve Veteran Involvement in Care, VA Portland Health Care System, Veterans Health Administration, United States Department of Veterans Affairs, Portland, OR, United States; ^2^School of Graduate Psychology, Pacific University, Hillsboro, OR, United States; ^3^Northwest ADHD Treatment Center, Portland, OR, United States; ^4^Wayne State University, Detroit, MI, United States

**Keywords:** mindfulness, substance use disorders, efficacy and safety, minoritized populations, addictive behavior

Substance Use Disorder (SUD) is a chronic condition impacting 176 million people worldwide (Castaldelli-Maia and Bhugra, 2022). Traditional Relapse Prevention focuses on psychoeducation and management of external triggers (Larimer et al., 1999); however, management of *internal* relapse triggers (e.g., craving, negative affect) remain underexamined. Up to 60% of individuals relapse after treatment completion (McLellan et al., 2000), suggesting traditional approaches are not effective in maintaining treatment gains.

Over the last two decades, third-wave cognitive behavioral therapies such as mindfulness- based interventions (MBIs) were developed in response to the short-comings of traditional SUD treatments. Distinct from second-wave therapies, the third-wave focuses on changing one's *relationship* to aversive internal experiences rather than changing the content of those states (Brown et al., 2011). In addition to managing external substance use cues like traditional SUD therapies, practices utilized in MBIs address internal substance use cues that result in relapse (Korecki et al., 2020). As such, MBIs show effectiveness across a variety of SUDs within various clinical contexts, greater reductions in substance use, and reduced cost compared to traditional treatments (Korecki et al., 2020; Félix-Junior et al., 2022; Cavicchioli et al., 2018).

Despite the effectiveness of MBIs for SUD, the majority have been validated on Non-Hispanic, White, males, with few studies reporting demographics of people with minoritized identities (Waldron et al., 2018). The term “minoritized” describes subpopulations made more vulnerable to health disparities because of their identity. For example, counties with a higher proportion of uninsured individuals or Black Americans are less likely to have SUD facilities that accept Medicaid, in which Black and Latinx patients are over-represented (Acevedo et al., 2018). Minoritized individuals are more likely to face systemic barriers to treatment, and show greater mistrust of healthcare institutions due to historical discrimination (Benkert et al., 2019). Additionally, minoritized populations have greater odds of substance dependence, treatment dropout, and increased rates of relapse (Saloner and Lê Cook, 2013). Given that MBIs are becoming more regularly implemented within the clinical milieu and few MBIs have been validated within minoritized populations, this Research Topic aims to increase our understanding of the relevance of MBIs within minoritized communities.

The six included articles encompass a broad range of research designs that enhance our understanding of MBIs among populations who have been historically marginalized and underrepresented in research. The first two articles are theoretical works focused on the benefit of MBIs in the role of stigmatization among minoritized populations with SUD, which poses a significant barrier to treatment engagement. The Moniz-Lewis article, *The Mindful Resiliency in Recovery Model: Empowering the Transcendence of Stigma*, proposes a novel framework to enhance engagement and effectiveness of MBIs for marginalized individuals through reducing self-stigma, and promoting resilience. MBIs can promote a non-judgmental stance toward stigma and bolster acceptance and self-compassion in moments when stigma is experienced. This in turn enhances resiliency and potentially boosts the efficacy of MBIs among marginalized populations. Similarly, Shank et al.'s article, *Substance Use During Pregnancy: The Role of Mindfulness in Reducing Stigma*, discusses the role of mindfulness in reducing bias among healthcare providers, in turn reducing stigmatization of pregnant people with SUD. The article purports that enhancing meta-cognitive processes through mindfulness can reduce provider bias by bringing awareness to automatic negative beliefs that may impact the quality of healthcare provided. Greater awareness of biases enables providers to respond to patients in a way that reduces judgement and increases engagement. These works highlight the importance of addressing stigma in SUD treatment, particularly among minoritized populations, and the mechanisms by which MBIs may reduce stigma and enhance treatment outcomes.

Two articles used a mixed-methods approach to evaluate MBIs among minoritized populations. Bautista et al. compared satisfaction with “Moment-by-Moment Women's Recovery,” among primarily Latinx women with SUD in residential treatment (*N* = 54) showing high to low trauma severity. Women with high trauma symptom severity reported lower treatment satisfaction at session two compared to women with low symptom severity, but no differences were found at session 11, indicating the importance of early retention strategies among minoritized women with SUD and co-occurring trauma. Machado et al. assessed the acceptability of Mindfulness-Based Relapse Prevention (MBRP), which has primarily been validated in high income countries, within eight SUD treatment centers in Sao Paulo, Brazil. MBRP demonstrated acceptability among patients (*n* = 140) providers (*n* = 24), and clinical managers (*n* = 7). However, adaptations to the structure of the original format were recommended to improve retention among individuals who experience greater adverse social impacts (e.g., houselessness). Lack of provider training was also identified as a barrier to implementing MBRP in community treatment facilities. Under the broader umbrella of addictive behaviors, Fernandez-Crespo et al. contributed an article outlining a study protocol for a randomized controlled trial of a MBI for problematic smartphone use in a sample of Spanish-speaking young adults. The authors suggest the development of effective primary care interventions for reducing the risk of developing smartphone addiction and alleviating symptoms associated with problematic use. Taken together, MBIs for addictive behaviors are acceptable among minoritized populations; however, additional efforts toward retention and intervention adaptation are warranted to bolster treatment outcomes.

Finally, a community-based study by Gawande et al. utilized qualitative analysis to explore the implementation of Mandela Yoga, a 12-week peer-led protocol among primarily Spanish-speaking men of color recently released from incarceration (*N* = 15). Individual interviews were conducted with one facilitator and one participant to explore elements of the intervention that most impacted participant SUD recovery. The intervention was feasible, and thematic analysis of interviews identified several of the most impactful elements of the intervention: 1) Breath and Mind-Body Connection Leads to Presence, 2) Consistency, 3) Peer Connection, and 4) Agency and Positive Action. Findings indicate that the benefits of Mandela Yoga extend beyond those of existing MBIs for communities of color.

Together, these articles highlight the importance of research exploring the relevance and efficacy of MBIs among various minoritized groups experiencing addiction. This collection points to the utility of MBIs in improving treatment access, engagement, and outcomes among historically marginalized groups. Future research on the development and implementation of culturally adapted MBIs may be a particularly promising avenue for addressing health disparities and systemic barriers to healthcare.
